# Approach to the Patient: The Evaluation and Management of Men ≥50 Years With Low Serum Testosterone Concentration

**DOI:** 10.1210/clinem/dgad180

**Published:** 2023-03-30

**Authors:** Mathis Grossmann, Channa N Jayasena, Bradley D Anawalt

**Affiliations:** Department of Medicine (Austin Health), The University of Melbourne, Melbourne, Victoria 3084, Australia; Department of Endocrinology, Austin Health, Heidelberg, Victoria 3084, Australia; Section of Investigative Medicine, Imperial College London, London SW7 2AZ, UK; Department of Medicine, University of Washington School of Medicine, Seattle, WA 98195, USA

**Keywords:** testosterone, hypogonadism, androgen deficiency, aging men, testosterone therapy

## Abstract

Although testosterone replacement in men with classic hypogonadism due to an identified pathology of the hypothalamic-pituitary-testicular axis is uncontroversial, the role of testosterone treatment for men with age-related declines in circulating testosterone is unclear. This is due to the lack of large, long-term testosterone therapy trials assessing definitive clinical endpoints. However, men ≥50 years of age, particularly those who have a body mass index >25 kg/m^2^ and multiple comorbidities, commonly present with clinical features of androgen deficiency and low serum testosterone concentrations. Clinicians are faced with the question whether to initiate testosterone therapy, a difficult dilemma that entails a benefit-risk analysis with limited evidence from clinical trials. Using a case scenario, we present a practical approach to the clinical assessment and management of such men.

Men with low serum testosterone concentrations can be characterized as having classic hypogonadism (sometimes called *organic* hypogonadism) due to an identifiable pathology or disorder of the hypothalamic-pituitary-testicular (HPT, gonadal) axis, or they can be characterized as eugonadal with suppression of the gonadal axis due to a systemic disorder or condition (1). A low serum testosterone due to suppression of the gonadal axis by a systemic disorder is analogous to the changes in thyroid function seen in euthyroid sick syndrome and is sometimes described as *functional hypogonadism*. Similar to euthyroid sick syndrome (or nonthyroidal illness), men with low serum testosterone due to suppression of the HPT axis by systemic disease might have underlying normal HPT function. Because the serum testosterone might normalize if the underlying disorder is treated and health restored, *eugonadal sick syndrome* or *nongonadal illness* might be more accurate terms than *functional hypogonadism* (2). These terms have the advantage of identifying the underlying gonadal axis as normal and not conflating *functional* with *reversible*; pathologies and disorders associated with classic hypogonadism are also potentially reversible (eg, hyperprolactinemia, iron overload syndromes). Classic pathologic hypogonadism is relatively uncommon with estimated lifetime prevalence <1% (3). Low serum testosterone due to suppression of the HPT axis is common and increases with age and body mass index (BMI) (4). Some experts use the term *late-onset hypogonadism* for functional hypogonadism in older men. The terms *functional hypogonadism* and *late-onset hypogonadism* suggest that testosterone treatment might be beneficial, a concept that has not been proven.

In the population-based European Male Ageing Study, the prevalence of the syndromic association of symptoms of hypogonadism plus low serum testosterone without an identifiable classic HPT axis pathology was determined in men aged 40 to 79 years (5). Symptoms of hypogonadism were defined as a constellation of low libido, decreased sexual function, and a low serum testosterone. The overall prevalence of this syndrome of androgen-deficiency-associated sexual symptoms plus low serum testosterone was 2.1%, and it increased from 0.6% in men 50 to 59 years of age to 5.1% in men 70 to 79 years. Obesity (BMI > 30 kg/m^2^) was associated with a 13-fold increased prevalence compared with normal weight. Likewise, the presence of ≥ 2 comorbidities was associated with a 9-fold increase in the prevalent syndrome of hypogonadism-associated sexual symptoms plus low serum testosterone. In the longitudinal follow-up, weight loss was associated with resolution of this syndrome in a significant percentage of the men (6).

The management of older men with low serum testosterone concentrations is hampered by the fact that many clinical features of androgen deficiency are nonspecific and similar to those associated with obesity or comorbid disease, conditions that are associated with low serum testosterone concentrations. In such men, the conundrum lies in determining the degree to which the (often nonspecific) clinical symptoms are predominantly due to hypogonadism or instead due to obesity and other chronic systemic disorders that suppress the HPT axis (ie, reverse causality). Of note, this assessment is probabilistic rather than deterministic. In many men, this assessment can only be validated during management, by monitoring the response to lifestyle measures, optimization of comorbidities, and in selected cases, a trial of testosterone treatment. However, a trial of testosterone therapy should only be considered when an older man has persistent symptoms suggestive of hypogonadism and reproducibly low serum testosterone concentrations after a minimum of 6-12 months of addressing reversible causes of a suppressed HPT, including vigorous lifestyle changes for overweight men. It is particularly important to use a standardized, harmonized total testosterone assay (if at all possible) when considering a trial of testosterone therapy.

In this article, we use a case-based approach to outline the assessment and management of men older than 50 years presenting with features of androgen deficiency and a low testosterone level, based on a review of the literature (PubMed), using the search terms “*male hypogonadism*,” “*testosterone deficiency*,” “*androgen deficiency*,” “*functional hypogonadism*,” “*late onset hypogonadism*,” as well as the clinical experience of the authors. We will focus on the diagnosis and management of possible androgen deficiency (Fig. 1). Fertility will not be discussed. Many of the principles and recommendations that we discuss are pertinent to men under age 50. However, we focus on men ≥50 years old in this manuscript because of the following: 1) the prevalence of low serum testosterone concentrations without identifiable HPT pathology increases significantly in men starting at age 40-50; 2) most of the high-quality epidemiological and randomized controlled studies of men with low serum testosterone concentrations without identifiable HPT pathology were performed in men ≥50 years old; 3) the diagnostic evaluation and management of men ≥50 years old differs from younger because a) evaluation for hypothalamic and pituitary disease with sellar imaging and evaluation for iron overload syndromes is often unnecessary in older men; b) infertility due to HPT dysfunction is less commonly important for men ≥50 years old; and c) long-term suppression of the HPT axis (iatrogenic androgen deficiency) is much more likely to occur with exogenous testosterone therapy in men ≥50 years old.

**Figure 1.**
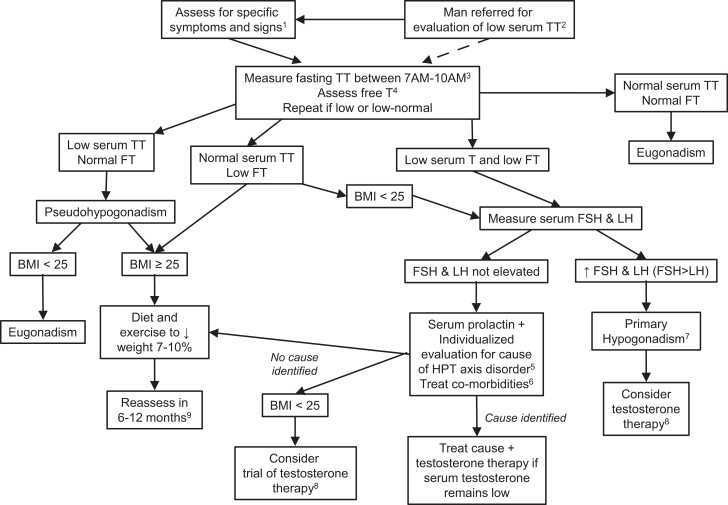
Approach to diagnosis and management of possible hypogonadism in men ≥50 years. ^1^Recent onset of declining libido and/or erectile function, new onset or tender gynecomastia; osteoporosis (see Table 1). ^2^Patients are often referred for consultation about low serum total testosterone (TT) that was measured in the absence of high clinical suspicion of hypogonadism. It is generally useful to repeat the measurement in this clinical setting. ^3^Measure TT when patient is at baseline health. Confirm low TT with a second fasting sample. At a minimum, the confirmatory measurement should be done on a fasting sample drawn between 7 and 10 Am. ^4^Assess serum free testosterone (FT) on confirmatory sample using an accurate method if the serum TT is 220-264 ng/dL (7.6-9.2 nmol/L) or if abnormalities in serum SHBG are suspected (see Table 2). Although some experts discourage the measurement of free testosterone given the limitations discussed in the manuscript and focus instead on interpretation of SHBG along with total testosterone (7), this approach is congruent with assessing serum free testosterone with an accurate method. ^5^Investigation for cause of hypothalamic-pituitary-testicular (HPT) disorder includes clinical assessment for Cushing syndrome, review of medications that affect the HPT axis and measurement of serum prolactin. Measurement of serum T4 might be useful if panhypopituitarism is suspected. Sellar imaging is indicated if serum prolactin is elevated. Evaluation of hyperprolactinemia also includes review of medications and measurement of TSH. Sellar imaging also indicated if there are symptoms of a sellar mass, serum LH is undetectable, or serum total testosterone is <150 ng/dL (5.2 nmol/L). ^6^This includes optimization of management of chronic disease, stopping medications that can suppress the HPT axis if possible (eg, opioids, glucocorticoids), evaluation and treatment of depression and sleep apnea. ^7^Consider karyotype for assessment for Klinefelter syndrome; offer testosterone treatment if there is no contraindication (including desire to conceive in the next 1-2 years). ^8^The decision to offer testosterone therapy should take into consideration the likelihood of benefit and the potential risk. The benefits are highest in men with lower testosterone concentrations and men with concordantly low total and free testosterone concentrations. A trial of testosterone therapy may be offered if the following conditions are met: 1) there are no contraindications; 2) there has been a discussion regarding potential benefits and risks of testosterone therapy and the uncertainties regarding long-term health outcomes; 3) patient-specific goals have been set; and 4) there is agreement that testosterone treatment will be stopped if these goals are not achieved. See text in manuscript. ^9^Reassessment of men with high BMI consists of assessment for symptoms and signs of hypogonadism and re-measurement of testosterone. If there are symptoms of hypogonadism and low serum testosterone after 6-12 months of lifestyle changes, then testosterone therapy may be considered. Medical therapy to achieve weight loss and/or bariatric surgery should be considered for men whose BMI remains >30 kg/m^2^ after 6 months of lifestyle changes. Abbreviations: BMI, body mass index; FT, free testosterone; HPT, hypothalamic-pituitary-testicular axis; TSH, thyroid stimulating hormone; TT, total testosterone; SHBG, sex hormone binding globulin.

## The Case

A 67-year-old man is referred for evaluation and management of possible hypogonadism. He reports that his libido has declined over the last 10 years, but his predominant sexual complaints are that his erections have gradually become less firm resulting in decreased sexual satisfaction. He reports fatigue and “feeling washed out” on most days. His wife sleeps in another room because he snores very loudly. She reports that he has occasional gasping for breath when sleeping. He reports low mood that has worsened since retirement 2 years ago. Comorbidities include obesity, type 2 diabetes mellitus (T2D) diagnosed 5 years ago, hypertension, and hypercholesterolemia. He is sedentary. He has a 30-pack-year history of smoking, but he quit smoking 5 years ago. He has no history of previous myocardial infarction or stroke. He was hospitalized briefly for treatment of a viral pneumonia about 10 weeks ago. His current medications include lisinopril, atorvastatin, metformin, and sitagliptin. He has never used glucocorticoids, opioids, or anabolic steroids. He reports no bruising tendency, weakness, headaches, or visual disturbance. He has fathered 2 children (now adults) without difficulty. He recalls normal puberty at “about the same time as his friends.” On physical examination, his blood pressure is 140/90 mmHg, weight is 99.3 kg (219 lb), and BMI is 31.3 kg/m^2^. He has normal body hair and lipomastia, but no palpable gynecomastia. His genital exam is normal (including testes that measure 20 cc each by Prader orchidometry). Pedal pulses are reduced. The remainder of the exam is normal, including cardiopulmonary, abdominal, skin, and neurological exam is normal.

Results from tests ordered by his primary care provider and performed on a nonfasting afternoon blood sample drawn 8 weeks ago demonstrated a serum total testosterone of 138.3 ng/dL (4.9 nmol/L) (normal range, 264-916 ng/dL or 9.16-31.8 nmol/L), a serum HbA1c of 8.1% and a serum hemoglobin of 14.5 g/dL (normal range, 13.5-17.5 g/dL). Liver function and kidney function tests were normal. Bone densitometry 6 months ago revealed a T score of −1.8 in the lumbar spine and −1.6 in the left total hip and femoral neck.

## How Should This Man Be Evaluated?

### Does This Man Have Clinically Evident Androgen Deficiency?

The first step is to determine his pretest probability of hypogonadism. There is significant overlap of the nonspecific symptoms of hypogonadism and depression. Because of his report of depressed mood and fatigue, the patient was assessed by the Patient Health Questionnaire-9 screening tool that screened negative for major depression.

Careful assessment for symptoms and signs that are more specific for hypogonadism is a key first step in the evaluation (Table 1). In the initial cross-sectional report from the European Male Ageing Study, 3 sexual symptoms (low libido, erectile dysfunction, and reduced morning erections) were the only symptoms (out of a total of 19 candidate symptoms) that were consistently associated with a low serum testosterone in a syndromic fashion; diminished libido had the highest odds ratio for a low serum testosterone concentration (5). Of note, this epidemiology study cannot determine causality. Because reduced sexual activity has been proposed to lower serum testosterone (8), the observed association of sexual symptoms with low serum testosterone concentrations in the European Male Ageing Study might reflect reverse causation. In the European Male Aging Study, the prevalence of these sexual symptoms was 25% to 35% even in men with a normal serum testosterone (5). His symptoms of fatigue and low mood are nonspecific and have low diagnostic value. They may be caused by systemic disease (*see below*). However, the constellation of symptoms of sexual dysfunction and low serum testosterone justify further evaluation for androgen deficiency.

**Table 1.** Clinical features of testosterone deficiency*^a^* in men

Specific	Very small testes (combined volume ≤8 cc)*^b^*
	Pubertal delay*^b^*
	Eunuchoid body proportions*^b^*
	Deficient male pattern body hair*^c^*
Suggestive	Decreased or low libido (particularly when confirmed by partner)
	Loss of early morning erections
	Vasomotor symptoms
	New-onset or increasing gynecomastia with breast tenderness
	Reduced testicular volume (volume 6-12 cc each testis)
	Unexplained anemia
	Poor semen quality and/or sperm concentration <15 million/mL
	Osteoporosis or unexplained osteopenia
Nonspecific	Fatigue
	Low mood
	Erectile dysfunction
	Reduced lean-to-fat mass

Some effects of testosterone deficiency (eg, reduced bone density, increased fat mass, vasomotor symptoms) might be predominantly mediated by concomitant deficiency of estradiol, a testosterone metabolite).

Seen only in men with prepubertal onset of hypogonadism; uncommon in older men without a diagnosis of classical hypogonadism.

Only in men with very longstanding, untreated hypogonadism.

The patient lacks more specific clinical signs of hypogonadism (Table 1), but these findings, such as new-onset or increased gynecomastia that is almost always characterized by breast pain and tenderness, deficient male pattern body hair (that is generally only seen in longstanding hypogonadism), hot flashes that might occur with acute onset of hypogonadism (eg, bilateral orchidectomy), and/or small (<15 cc) testes are not common in hypogonadal middle-aged or older men (9). Although small testes are an uncommon finding in older men with newly diagnosed hypogonadism, Klinefelter syndrome, the most common cause of classic organic hypogonadism, is typically characterized by very small testes (≤4 cc each), is underdiagnosed (by 50% or more), and might not be diagnosed until middle or older age (3).

The patient does not have unexplained anemia that might suggest hypogonadism. His osteopenia is consistent with, but not specific for sex steroid deficiency. Observational and experimental studies consistently suggest that bone health is typically compromised at serum total testosterone concentrations of <200 ng/dL (6.9 nmol/L) (10).

His initial assessment identifies comorbidities that could be partially responsible for his clinical presentation. Reduced pedal pulses and erectile dysfunction indicates likely atherosclerosis and increased cardiovascular risk (11). The clinical history suggests uncontrolled sleep apnea that could be the primary cause of his fatigue and suppressed HPT axis and a factor in his erectile dysfunction (12). Of note, hypoxemia in older men with sleep apnea can be associated with increases in hematocrit (13) and thus could mask testosterone deficiency–associated anemia in this man.

There are no clinical features suggesting classic organic causes of hypogonadism, including Klinefelter syndrome, hypopituitarism, pituitary mass effect, or endogenous Cushing syndrome, and he is not taking medications that can suppress the HPT axis, such as opioids or glucocorticoids. Moreover, there is no history of common risk factors for primary and secondary hypogonadism such as testicular trauma, radiation to the pelvis, chemotherapy, significant head trauma, or radiation to the head or neck. In summary, his clinical probability of classic pathologic hypogonadism is low to moderate. His symptoms might be due to depression (with a false negative result in the Patient Health Questionnaire-9), atherosclerotic disease of the penile vasculature, obesity, sleep apnea, or some combination of these causes. Because the man has had a very low serum testosterone measured, it is incumbent to exclude hypogonadism with an appropriate evaluation (Fig. 1).

### How Should the Clinical Suspicion of Androgen Deficiency Be Assessed Biochemically?

Reproducibly low serum total testosterone concentrations measured by an accurate testosterone assay on a sample drawn in the early morning (7-10 Am) in the fasting state are a cornerstone of the diagnosis of male hypogonadism (9, 14). It is optimal to use a total testosterone assay that has been validated by an accuracy-based standardization or quality control program with a normal range of 264-916 ng/dL (9.16-31.8 nmol/L) in healthy, young men (14, 15). Use of such an assay facilitates application of findings of the T-Trials (16), the largest randomized controlled trial (RCT) comparing testosterone vs placebo with clinically important outcomes in men with low serum testosterone concentrations, and the soon-to-be-reported TRAVERSE study (17), a large RCT with cardiovascular outcomes; these trials used a standardized, harmonized total testosterone assay with this normal range (16, 17).

If the initial serum total testosterone is low, confirmation is required. Without compelling evidence (eg, specific clinical features, such as very small testes and high serum gonadotropin concentrations), androgen deficiency should not be diagnosed with a single low testosterone concentration because of significant diurnal and day-to-day variation (14). Up to 30% of older men with a low serum testosterone concentration have a normal value on repeat testing (18). Overnight fasting increases serum testosterone by 9% to 16% and reduces the significant diurnal variability of serum testosterone concentrations (19). Most experts prefer to err in underdiagnosing very mild hypogonadism in older men over misdiagnosing eugonadal men as hypogonadal. The benefit of testosterone therapy on vitality and bone (and likely other parameters) is related to the change from baseline serum testosterone in older men; men with lower serum baseline testosterone concentrations are more likely to benefit from normalization of serum testosterone (16, 20). Therefore, borderline low serum testosterone concentrations or multiple serum testosterone measurements that are discordant (eg, one result that is slightly low and one that is normal) should be interpreted cautiously and generally not as confirmatory evidence of hypogonadism (14).

## Back to the Case

Although this man's initial serum total testosterone concentration was very low, he had a recent severe illness, (hospitalization for pneumonia 2 weeks prior to the initial testosterone measurement). Any significant acute systemic illness or flare of chronic systemic illness can transiently suppress the HPT axis and serum testosterone. Assessment for hypogonadism should not be done in men who are acutely unwell (and below their baseline health) or during hospital admission. Although there are no prospective data on the time frame of HPT axis recovery after an acute systemic illness, clinical experience indicates that the time to recovery is inversely related to the severity and duration of the illness. Because making a diagnosis of hypogonadism is not urgent, we recommend deferring testosterone measurements until the patient has recovered to their baseline health for at least 2 months. In patients with a protracted systemic illness lasting more than a few weeks, serial measurements of testosterone over several months are helpful; a pattern of increasing serum testosterone suggests that the HPT axis might fully recover.

In our patient, the initial measurement of serum testosterone was done on a nonfasting afternoon sample. Serum testosterone has a circadian rhythm with highest concentrations in the morning, and the normal range of serum testosterone is based on early morning blood samples. Although this circadian rhythm might be attenuated in many older men (21), male hypogonadism should be diagnosed based on an early morning testosterone concentration. Lastly, this man's low serum testosterone was not confirmed with a second measurement.

### Serum Free Testosterone Assessments

Accurate assessment of serum free testosterone might be helpful when serum total testosterone concentration approximates the lower limit of normal or when abnormalities in sex hormone binding globulin (SHBG) are suspected (Table 2; Fig. 1). A fasting early morning total testosterone concentration of >350 ng/dL (12 nmol/L) measured by a reliable assay is generally consistent with a normal serum free testosterone (22) and typically does not require assessment of free testosterone except in men with suspected or proven high SHBG concentrations (*see below*). The most common causes of the constellation of a low serum total testosterone, low serum SHBG, and normal free serum testosterone (ie, *pseudohypogonadism*; Fig. 1) are obesity and T2D (Table 2) (23).

**Table 2.** Causes of high and low circulating SHBG

Decreased SHBG	Obesity
	Diabetes mellitus with poor glycemic control
	Androgens, glucocorticoids, and some progestins
	SHBG gene polymorphisms
	Hypothyroidism (untreated)
	Acromegaly (untreated)
	End-stage liver cirrhosis with severe synthetic dysfunction
	Nephrotic syndrome
Increased SHBG	Aging
	SHBG gene polymorphisms
	Chronic high alcohol consumption
	Some anticonvulsants (eg, phenobarbital)
	Estrogens
	Untreated or undertreated HIV infection (a direct hepatic effect)
	Liver disease (other than end-stage cirrhosis)
	Hyperthyroidism (untreated)
	Severe energy deficit (reduced caloric intake and/or excessive exercise)*^a^*

Abbreviation: SHBG, sex hormone binding globulin.

Friedl et al. *J Appl Physiol*. 2000;88(5):1820-30. doi:10.1152/jappl.2000.88.5.1820

There are no well-validated, outcome-based normal reference ranges for serum free testosterone concentration in older men (24). In addition, there is wide variation in the quality and the various methods used to measure or calculate serum free testosterone concentrations. Direct methods of measurement of free testosterone are inaccurate and should not be used (14).

A recent study (25) in 145 healthy nonobese men aged 19 years or older using a standardized method with mass spectrometry after equilibrium dialysis has reported a reference range for free testosterone of 66 to 309 pg/mL (229-1070 pmol/L); further validation is required, and equilibrium dialysis is currently not widely available (25). Equilibrium dialysis is the gold standard measure of free testosterone, but its methodology is complex and expensive, so it has limited availability worldwide. In practice, free testosterone is usually calculated by empiric formulae that have some pitfalls, but generally correlate reasonably well with values determined by equilibrium dialysis (24). There is some controversy about the best formula for estimating free testosterone concentrations. The Vermeulen formula is the most commonly used formula in clinical practice and trials, and it correlates reasonably well with measurement by equilibrium dialysis (24). If a validated, accurate and harmonized total testosterone assay (with a normal range of 264-916 ng/dL or 9.16-31.8 nmol/L) is used to measure serum total testosterone, then a calculated serum free testosterone provides a reasonable estimation of serum free testosterone. Assuming these conditions are met, then a lower limit of normal of 70 to 80 pg/mL pg/mL (242-277 pmol/L) is likely to be clinically reasonable. Using the limit of normal of 70 to 80 pg/mL pg/mL (242-277 pmol/L) that is higher than the lower limit of normal of 66 pg/mL (229 pmol/L) demonstrated by the above study by Jasuja (25) helps to limit the overdiagnosis of hypogonadism to men who are not likely to benefit from typical replacement dosages of testosterone therapy.

A free testosterone result is most useful in the assessment of a man with intermediate pretest probability of hypogonadism, a condition associated with low serum SHBG and a serum total testosterone that is moderately low to low-normal (ie, 200-275 ng/dL or 6.94-9.55 nmol/L). Some experts discourage the measurement of free testosterone because of the limitations discussed above, and these experts recommend focusing on the interpretation of total testosterone concentrations in conjunction with SHBG concentrations (7). However, this approach is congruent with assessment of serum free testosterone with an accurate method. Overall, there is no consistent evidence that free testosterone concentrations are a better measure of androgen status than total testosterone (26, 27). Further studies to help establish more accurate and uniform methods of assessing free testosterone concentrations and to determine its clinical utility for the diagnosis of hypogonadism are needed. For further discussion about the debate over the clinical value of free testosterone and the accuracy of its calculation/measurement, there are several recent reviews (24, 28, 29).

Some hypogonadal men with markedly elevated serum SHBG concentrations may have normal or even high serum total testosterone concentrations. Significantly elevated serum SHBG concentrations occur most commonly in the setting of anti-epileptic treatment, chronic liver disease, or chronic daily ingestion of large quantities of alcohol (even in the absence of significant elevation of serum hepatic transaminases) (Table 2) (30, 31). Marked SHBG elevations leading to misleadingly normal or even high total testosterone are relatively uncommon, whereas pseudohypogonadism in men with diabetes and/or obesity is very common.

## Back to the Case

Measurement of serum total testosterone concentrations was repeated twice 3 months later after the patient had returned to baseline health. The measurements were performed on fasting blood samples drawn 3 weeks apart in the early morning (7-10 Am): 236 ng/dL (8.2 nmol/L) and 245 ng/dL (8.5 nmol/L) (reference range, 264-916 ng/dL and 9.16-31.8 nmol/L; the assay was validated and harmonized by the United States Centers for Disease Control); SHBG 22.7 nmol/L (reference range, 10-60 nmol/L); calculated free testosterone (using the Vermeulen formula (32)) 56 pg/mL (194 pmol/L) (reference range, 66-272.3 pg/mL and 229-710 pmol/L).

### What Additional Diagnostic Evaluation Would Be Helpful in an Older Man With a Low Serum Testosterone Concentration That Has Been Appropriately Confirmed?

We have now confirmed that this patient has low serum testosterone concentrations. Does he have nongonadal illness or hypogonadism? The first step is the determination of serum gonadotropin concentrations. Elevated serum luteinizing hormone (LH) and follicle-stimulating hormone (FSH) concentrations would be consistent with primary hypogonadism. Primary hypogonadism increases to 2% to 7% prevalence in men over age 70; age over 70 is associated with a progressive decline of serum testosterone concentration and a rise of serum LH concentration (to >15 IU/L) in many men (33, 34). If this man has elevated FSH and LH concentrations, then he is hypogonadal. If he has low or normal serum gonadotropin concentrations, then he should be evaluated for causes of hypothalamic and pituitary dysfunction. To minimize phlebotomies and expedite the evaluation, we recommend ordering early morning, fasting serum total testosterone, SHBG, and gonadotropins at the first clinic visit.

Using serum gonadotropins as a pivot in the evaluation of possible hypogonadism is very useful. Although many causes of hypogonadism may affect the central (hypothalamus and/or pituitary) and peripheral (testes) components of the HPT axis, the serum gonadotropins indicate the site of the principal dysfunction. Primary hypogonadism defined by low testosterone concentrations and elevated gonadotropins is generally irreversible in older men, whereas many causes of secondary hypogonadism might be reversible (4). Thus, affirmation of elevated serum gonadotropins (FSH > LH) clinches the diagnosis of primary hypogonadism in a man with symptoms and/or signs suggestive of hypogonadism and a low serum testosterone concentration.

Although classic pathologic causes of hypogonadism have been described as *organic* and potentially reversible, *nonclassic* causes of hypogonadism such as obesity have been described as *functional*; we recommend classifying hypogonadism as primary or secondary and potentially reversible or irreversible. Klinefelter syndrome is an example of irreversible primary hypogonadism. Very high BMI (usually ≥40 kg/m^2^) (for review see (35)), severe systemic disease, endogenous Cushing syndrome, endogenous hyperprolactinemia, and treatment with corticosteroids, opioids, and anti-dopaminergic medications are common causes of potentially reversible secondary (or combined secondary and primary) hypogonadism. This typology of potentially reversible or irreversible primary and secondary hypogonadism is informative and clearer and more useful than *organic* and *functional*.

## Back to the Case

His serum FSH was 4.1 IU/L (reference range, 1.0-7.0 IU/L), and LH was 3.2 IU/L (reference range, 1.0-8.0 IU/L).

### What Additional Diagnostic Evaluation Is Necessary to Exclude Classic Organic Hypothalamic-Pituitary Pathology in a Man With Low Serum Total Testosterone and Normal Serum Gonadotropin Concentrations?

Most middle-aged and older, obese men with a low testosterone will have secondary hypogonadism with normal serum gonadotropin concentrations (33). Men with secondary hypogonadism should be clinically assessed for Cushing syndrome and sleep apnea. Evaluation for identifiable hypothalamic and pituitary pathology should be individualized because the yield is typically low. In men ≥50 years old with low serum testosterone and normal gonadotropin concentrations, clinical experience suggests that the probability of classic organic hypothalamic-pituitary pathology is inversely related to BMI, age, number of comorbidities, and testosterone concentration (14). In older, obese men, pseudohypogonadism due to obesity is the most common cause for the biochemical constellation of low serum total testosterone (due to low SHBG), normal serum free testosterone, and normal gonadotropins, reflecting a eugonadal state. In secondary hypogonadism due to structural or genetic hypothalamic-pituitary disorders, gonadotropins are often low or undetectable.

In the absence of clinically suspected hypothalamic-pituitary disease, measurement of serum prolactin is the only diagnostic test that is routinely recommended for evaluation of causes of secondary hypogonadism of men ≥50 years. Men with secondary hypogonadism and clinical or biochemical findings suggestive of a sellar mass or hypothalamic-pituitary disease, an elevated serum prolactin concentration, a very low serum total testosterone concentration, or serum gonadotropin concentration below the lower limit of normal should undergo sellar imaging. There is no well-defined threshold of total testosterone concentration for determining when to perform sellar imaging. The published literature, summarized by Hirsch et al (36), included 313 middle-aged and older men (mean age 57 years) with sexual symptoms and a mean total testosterone concentration of 190 ng/dL (6.6 nmol/L). Only 6 patients (1.9%) had a macroadenoma, all of whom had total testosterone concentrations of 104 ng/dL (3.6 nmol/L) or less. The Endocrine Society guidelines recommend pituitary imaging for men with symptoms of tumor mass effect (eg, visual impairment, visual field defect, or new-onset headache), panhypopituitarism, persistent hyperprolactinemia, or a serum total testosterone <150 ng/dL (5.2 nmol/L) (14). Of note, if prolactin is not elevated, a pituitary lesion has to be relatively large to cause hypogonadotropic hypogonadism, and therefore sellar computed tomography (CT) has sufficient sensitivity. A sellar CT involves a modest radiation exposure, but it is less likely to detect an incidental pituitary microadenoma. This lower sensitivity to detect a microadenoma might be an advantage compared to MRI scanning because nonsecretory pituitary microadenomas are common and are typically clinically inconsequential in older men (37, 38).

## Back to the Case

There were no specific symptoms (easy bruisability or proximal muscle weakness) or signs (diffuse ecchymoses, broad purple striae, inability to rise without using arms to assist) of Cushing syndrome; no biochemical testing for Cushing syndrome was done. Serum prolactin was normal. Because he was assessed to have very low probability of a sellar mass, pituitary imaging was not performed. The patient underwent a polysomnogram that confirmed moderately severe obstructive sleep apnea.

## What Treatment Would You Recommend?

This patient has nonspecific symptoms and signs of androgen deficiency, and his serum total testosterone is modestly reduced. The diagnostic evaluation has excluded classic pathology of the HPT axis. His symptoms, signs, and low serum testosterone (with normal serum gonadotropin) concentrations suggest HPT suppression due to obesity, untreated sleep apnea, and T2D with suboptimal glycemic control. A high percentage of older, obese men with low serum testosterone with normal serum gonadotropin concentrations will remit to normal serum testosterone concentrations, particularly with weight loss (4, 39). Initial treatment of our patient should include 1) implementation of lifestyle measures and pharmacotherapy to optimize weight and comorbidities (including cardiovascular risk) and 2) initiation of phosphodiesterase type 5-inhibitor (PDE5-inhibitor) and continuous positive airway pressure (CPAP) therapy to address his principal concerns of erectile dysfunction and fatigue. This holistic approach will provide general health benefits and has the potential to reverse his hypogonadism.

### What is the Evidence for Lifestyle Measures?

In middle-aged and older men not selected for a low serum testosterone, moderate exercise (eg, 90 minutes thrice weekly) improves sense of well-being, sarcopenia/physical function (40), bone mass, and cardiovascular risk factors (41), and might additively improve erectile function with PDE5-inhibitor treatment (42).

In a 2013 meta-analysis of studies of adult men with a BMI of 30 to 39.9 kg/m^2^, a hypocaloric diet leading to a 9.8% loss of body weight was associated with an increase in mean serum total testosterone concentration of 83 ng/dL (95% CI, 48-117) (2.87 nmol/L; 95% CI, 1.68-4.07), representing increases ranging from 10% to 30% of the baseline serum testosterone. Younger age and magnitude of weight loss were associated with the increment in serum testosterone (43). The studies included in the meta-analysis were small (generally <50 men), short term (<12 months), and predominantly recruited men <50 years of age. Furthermore, some men had normal baseline testosterone concentrations, and the overall effect was largely driven by a single study. In a 2016 RCT of a hypocaloric diet and testosterone vs placebo of obese men (baseline median age 53 years; BMI 37 kg/m^2^; serum total testosterone 202 ng/dL [7.0 nmol/L]), the mean weight loss of 9.1% achieved with the hypocaloric diet alone was associated with a 52 ng/dL (1.8 nmol/L) increase in total testosterone (a 20% increase from baseline) (44). Neither weight loss nor serum total testosterone increase were maintained after supervised weight loss intervention ended (45). In a 12-month RCT of frail obese men ≥65 years with a baseline serum total testosterone concentration of 211 to 274 ng/dL [7.3-9.5 mmol/L], weight loss by diet (mean loss 10.1 kg) and diet plus exercise (mean loss 9.1 kg) increased serum testosterone concentrations by 21.0 ng/dL (0.73 nmol/L) and by 49.9 ng/dL (1.7 nmol/L), respectively, without any changes in serum calculated free testosterone (46). The diet plus exercise group also had improved physical function (46). It is uncertain whether the increases in serum testosterone play a role in improved outcomes seen with weight loss. For example, some studies suggest that improved sexual function after bariatric surgery more closely correlates with the amount of weight loss than the rise of serum testosterone (47).

Thus, in addition to providing general health benefits, there is evidence that lifestyle changes might increase mean serum testosterone by 10% to 30% that might normalize testosterone concentrations in our patient and might improve many of his symptoms that overlap with hypogonadism. However, the data stem mostly from cohort studies of supervised lifestyle interventions, and these studies had small numbers of participants that were typically younger (30-50 years of age), relatively short follow-up (<1-2 years), and variable methodologies in the assessment of serum testosterone concentrations. Some evidence suggests that lifestyle interventions might be less successful normalizing serum testosterone concentrations and improving function in older, frailer men (46). The degree to which the increase in serum testosterone is responsible for the improvement seen with androgen deficiency-like features (eg, erectile function, physical performance) is not certain. Whether concomitant testosterone treatment improves the efficacy of lifestyle interventions requires further study, but preliminary evidence suggests that testosterone therapy preserves lean mass compared to placebo during caloric restriction (44, 48).

In summary, the single most important intervention for our patient and many older men with a high BMI and low serum testosterone concentration might be lifestyle changes (increased exercise, caloric restriction, and improved diet) to reduce weight.

### What Is the Evidence for Optimization of Relevant Comorbidities?

Relevant comorbidities in this case include obstructive sleep apnea and suboptimally controlled T2D. Obstructive sleep apnea is commonly associated with a low serum testosterone concentration. Although some studies suggest that obesity that is common in men with obstructive sleep apnea might be the primary determinant of reduced testosterone, other studies refute this conclusion (12, 49, 50). Whether CPAP therapy is associated with changes in serum testosterone remains unresolved, due to the lack of definitive studies (51, 52). Effective and regular use of CPAP, however, improves daytime somnolence, quality of life, and may improve erectile dysfunction (53).

Observational studies suggest that about 50% of men with T2D have modest reductions in serum total testosterone concentrations (mean concentrations typically ∼230-288 ng/dL [∼8-10 nmol/L]), and serum total testosterone concentrations are inversely associated with insulin resistance independently of BMI (54, 55). Given his suboptimal glycemic control and increased cardiovascular risk, initiation of agents with proven cardiovascular benefits is indicated, such as glucagon-like peptide-1 (GLP-1) agonists and/or sodium-glucose cotransporter-2 (SGLT2) inhibitors, and these agents also promote weight loss. Moreover, there is some evidence that (perhaps due to weight loss effects) GLP-1 agonists may increase serum total testosterone by 20% to 30% and lower the incidence of moderate or severe erectile dysfunction (56–58). Although there are no published studies that have examined the effects of SGLT2 inhibitors on the HPT axis, anecdotal evidence suggests that SGLT2 inhibitors might increase the risk of erythrocytosis during concomitant testosterone treatment (via reduced plasma volume and possible indirect effects on erythropoietin) (59).

## Back to the Case

The evidence was discussed with the patient. He agreed to implement lifestyle measures and was referred to a dietician and exercise physiologist with a target weight loss of 5% to 10% that might increase his serum testosterone into the normal range. However, despite his initial enthusiasm and hiring a personal coach, he was only able to maintain modest dietary and exercise changes. PDE5-inhibitor treatment was progressively titrated to maximum dose, with modest improvements in his erectile function. CPAP therapy was commenced and optimized with improvements in fatigue; repeat polysomnography demonstrated resolution of significant apneic episodes. He was prescribed a weekly GLP-1 receptor agonist, but he was unable to tolerate a low dosage of GLP-1 receptor agonist due to significant gastrointestinal side effects. SGLT2 inhibitor treatment was initiated.

Twelve months later, he had lost 2 kg (now 97.3 kg, 214 lb), and his HbA1c had decreased to 7.3%. A fasting early morning blood sample demonstrated a total serum testosterone (216 ng/dL; 7.5 nmol/L; ) and a low calculated free testosterone (51.6 pg/mL; 179 pmol/L). A second blood sample yielded similar results. Both total testosterone measurements were done by a validated, harmonized assay. Although his energy has improved, he still reports low motivation and intermittent low mood. He reports improved erectile function, but libido, tumescence, and overall sexual function remains less than satisfactory. The patient wishes to discuss the potential benefits and risks of testosterone therapy.

### What Are the Benefits of Testosterone Treatment?

In men with classic organic hypogonadism, testosterone replacement ameliorates the clinical features of hypogonadism (except fertility) although the evidence is limited to uncontrolled studies (60). In the following section we will focus on double-blind placebo-controlled RCTs of testosterone treatment in older men with low serum testosterone and no classic cause of HPT axis pathology. Benefits and risks are summarized in Table 3.

**Table 3.** Potential risks and benefits of testosterone treatment for men ≥50 years old with reduced serum testosterone

Potential benefits	Sexual function	Improved libido, sexual satisfaction, and modest improvement in erectile function
	Vitality	No effect
	Mood	Minimal or small benefit
	Cognition	No confirmed effect using several validated scores
	Physical function	Improved 6-minute walking test
	Glucose metabolism	Reduced or delayed incidence of type 2 diabetes in men with high risk of type 2 diabetes*^a^*
	Bone	Improvement in areal and volumetric bone mineral density and strength of lumbar and femoral region, improved microarchitecture*^a^*
	Unexplained anemia	Reduced incidence or ameliorated
Potential risks	Suppression of endogenous testosterone production and spermatogenesis	Universal—affects all men taking exogenous testosterone
	Erythrocytosis	Common adverse effect
	Urinary symptoms	Theoretical risk of worsening, but no strong evidence in men who do not have baseline severe symptoms
	Prostate cancer	No evidence of increased de novo cancer; increased growth high-grade or metastatic prostate cancer; increased risk of prostate biopsies and overtreatment of prostate cancer if prostate cancer screening is done as part of monitoring testosterone therapy
	Cardiovascular	No adequately powered published randomized controlled trials (RCTs) of testosterone with cardiovascular outcomes; no evidence for increased risk in existing RCTs with treatment duration ≤1year*^b^*
	Lipids	Modest (5%-15%) reduction in serum high density lipoprotein (HDL) concentration
	Blood pressure	No consistent effects identified

Pharmacological effect that is reported in men with low and low-normal serum testosterone concentrations.

The evidence for cardiovascular effects of testosterone is limited, but it does not support a large effect on cardiovascular outcomes (see manuscript).

#### Sexual function, energy/vitality, cognition, and mood

The T-Trials, the most definitive trial of older men with low serum total testosterone to date, recruited 790 men (who were similar to our patient) without known hypogonadism and a serum total testosterone concentration <275 ng/dL; (<9.54 nmol/L). The mean age was 72 years, 62.9% were obese, and mean baseline serum total testosterone was 232 ng/dL (8.0 mmol/L). About 10% of men were taking PDE5-inhibitor treatment at baseline (16). In the primary analysis among men with self-reported low libido and a partner willing to have sexual intercourse at least twice a month, testosterone treatment increased sexual activity at 3, 6, and 9 months, but this declined at 12 months (16). In subsequently reported more detailed analyses (61), testosterone treatment improved 10 of 12 measures in sexual activity (including sexual desire, activity, and overall satisfaction) with standardized mean differences (SMD) up to 0.45 (SMDs of 0.2-05 represent a modest treatment effect). The average increase in erectile function (2.64 in Erectile Function domain of the International Index of Erectile Function score [IIEF-EFD]) was below the clinically significant threshold of ∼ 4.0 (16). However, this threshold of 4.0 is for men with erectile dysfunction; the T-Trials did not include erectile dysfunction as an entry criterion and likely included men with a broad range of erectile function. Many men who do not meet the criteria for erectile dysfunction for clinical trials might consider an increase of 2.64 as clinically significant. In agreement with findings of the T-Trials, a meta-analysis of RCTs of 2298 men with a mean age of 60 years and a serum testosterone <346 ng/dL (<12 nmol/L) reported that testosterone treatment increased the IIEF-EFD score by 2.3, but this effect was attenuated in men with T2D and obesity (62). PDE5-inhibitors are more effective than testosterone therapy for treatment of erectile dysfunction in older men with low serum testosterone concentrations, and the added value of testosterone treatment in men receiving optimized PDE5-inhibitors treatment requires further study (63).

In the T-Trials, although testosterone therapy had no significant effects on vitality and mood in the primary analyses that focused on the subgroup of men with reduced vitality and mood at baseline, in secondary analyses, there was a small benefit on vitality and mood seen in the entire group of men randomized in the T-Trials. There was no measured effect on cognitive function, but cognitive function tests used in the trial might have been too insensitive (16). Overall, in men meeting the inclusion criteria of the T-Trials, testosterone treatment improves most aspects of sexual function and might lead to small improvements in vitality, mood, and depressive symptoms. Numerous tools have been utilized to measure the effects of testosterone on quality of life; a recent meta-analysis suggested a potential benefit, but the effect size was small, with low-to-moderate-certainty evidence (64).

#### Muscle strength and physical function

Increased lean mass (by about 1.6 to 4.7 kg) is one of the most consistent effects of testosterone treatment (65, 66), and in older men with mobility limitations, testosterone improves leg-press strength and stair-climbing power (67). In the T-Trials, testosterone treatment improved walking distance (assessed by the 6-minute walking test) and improved self-reported walking ability, but it did not reduce the risk of falls (68).

#### Glucose metabolism

Testosterone therapy reduces fat mass by about 1.6 to 4.3 kg (65, 66) but does not consistently improve measures of insulin resistance or glycemic control in men with low serum testosterone and established T2D (69). However, testosterone treatment has been reported to reduce the incidence of T2D in men with low to low-normal serum total testosterone concentrations and high risk of T2D (70). The differences in glycemic outcomes between men with established T2D (69) and those with prediabetes (70) suggests that testosterone treatment might be more effective if given early (ie, to high-risk men with insulin resistance driven by metabolically adverse changes in body composition), rather than men with longstanding T2D, who might have irreversible beta islet cell dysfunction. Further studies are needed to address this hypothesis.

#### Bone health

A meta-analysis of RCTs of using dual-energy x-ray absorptiometry (DXA) reported that testosterone treatment increased lumbar spine areal bone mineral density (BMD) (by 3.7% compared to placebo), while there was no significant increase in femoral areal BMD (65). A recent large study of 600 men, however, reported that testosterone treatment over 2 years was associated with increased femoral areal BMD (71). Moreover, studies, including a substudy of the T-Trials (72), using quantitative CT and high resolution–peripheral quantitative CT have reported that testosterone treatment increases cortical and trabecular volumetric bone density and estimated bone strength (71, 72). Men in these studies were not selected based on low bone density or osteoporosis (71, 72), and the effects of testosterone treatment on bone architecture, bone strength or incident fracture have not been systematically studied in men with low serum testosterone concentrations and low bone density or osteoporosis.

#### Erythropoiesis

Of the subset of men in the T-Trials who had baseline unexplained anemia (*n* = 126, baseline hemoglobin 10.0-12.7 g/dL), 50% of the testosterone-treated men reached the primary endpoint, an increase in hemoglobin concentration of 1.0 g/dL (an increment reported in previous anemia trials to have a positive effect on quality of life), compared with 15% randomized to placebo (adjusted odds ratio [OR] 31.5; 95% CI, 3.7-277.8; *P* = .002) (73).

### What Are the Potential Harms of Testosterone Treatment?

Erythrocytosis (hematocrit >54%) is the most common adverse effects of testosterone treatment in older men, with a relative risk of 8.14 [95% CI: 1.87, 35.40] in a meta-analysis (74). However, whether testosterone-induced erythrocytosis is associated with adverse (cardiovascular) outcomes is not known.

An additional common risk of androgen therapy is to suppress the HPT axis (75) and the suppression may persist for many months to years after long-term use. However, short-term use of 6 to 12 months is unlikely to cause dependence or long-term suppression; in this setting, recovery of the HPT axis typically occurs within 1 to 3 months (76). In addition, most men who are prescribed testosterone for nonspecific symptoms will quit testosterone therapy within 1 year (77, 78). The risk of persistent withdrawal or hypogonadism symptoms is unlikely to occur after a testosterone therapy trial of 1 year or less, and the patient is likely to stop therapy within 1 year if there is no benefit. However, longer courses of testosterone therapy may lead to more prolonged suppression of the male gonadal axis, potentially increasing the risk of re-prescribing testosterone; a recent follow-up of the T4DM study, concluded that after stopping 2 years of long-acting testosterone undecanoate treatment, full reproductive hormone recovery might take longer than 12 months to complete (75).

#### Cardiovascular health

Effects of testosterone treatment on cardiovascular outcomes remain unknown because there are no published long-term, placebo-controlled RCTs of testosterone therapy and cardiovascular outcomes as a primary outcome. The first such study, TRAVERSE (ClinicalTrials.gov Identifier: NCT03518034) has completed enrollment (*n* > 5200 men), and the findings of this study with ∼5-year follow-up are expected to be released in 2023; the study was fully recruited and was not ended early for prespecified cardiovascular outcomes (17); see https://www.clinicaltrials.gov/ct2/show/NCT03518034 (accessed January 2023). In some observational studies, higher endogenous serum testosterone concentrations have been associated with a decreased risk of cardiovascular events (79), but higher endogenous serum testosterone might be a marker of better overall health and fewer major comorbidities. Retrospective studies of testosterone treatment have suggested either reduced risks of cardiovascular events (80), neutral effects (81), or increased risks (82); such retrospective studies have significant methodologic shortcomings, including confounding by indication (ie, preferential treatment of healthier men) and time-related biases (79). Clinical trials of testosterone treatment have been mixed with 1 study in older men with mobility limitations reporting an increased risk of cardiovascular events (83) while there was no increase in a similar trial in frail older men (84). A total of 17 RCTs shared their individual patient data (*n* = 3431) to the Testosterone Efficacy and Safety Consortium (TestES) to report mortality and cardiovascular endpoints (85). Eighteen deaths (6 in testosterone- and 12 in placebo-treated men), and 230 cardiovascular events (120, testosterone; 110, placebo) were reported, without any significant differences between treatment groups. However, this analysis is limited by the short duration of constituent studies (mean 9.5 months) and exclusion of patients at high risk of cardiovascular disease by some studies (85). Although there was no increase of cardiovascular events in the T-Trials, a T-trial substudy reported, compared to placebo, a greater increase in coronary artery noncalcified plaque volume with testosterone treatment (86). However, groups were unbalanced for baseline plaque volumes, and the prognostic importance of the observed change in plaque volume is unclear. Moreover, in a 3-year RCT of men aged 60 years or older with baseline serum testosterone concentrations ranging from 100 to 400 ng/dL (3.5-13.9 nmol/L), testosterone treatment did not affect the change from baseline in coronary artery calcium score or common carotid artery intima-media thickness (87). Of note, most recent larger clinical trials have excluded men at high risk of cardiovascular events (16, 70). Whether testosterone treatment increases venous thromboembolic risk remains unresolved (88, 89). The United States Food and Drug Administration requires a testosterone products label warning of increased risk of myocardial infarctions, strokes, and venous thromboembolic events as a possible consequence of testosterone therapy. The 2018 Endocrine Society guidelines recommend against testosterone therapy in men with uncontrolled heart failure, myocardial infarction, or stroke within the last 6 months, or with thrombophilia (14).

#### Prostate health

Testosterone therapy increases serum prostate specific antigen concentrations in hypogonadal men to that of age-matched controls. Although there is no evidence that testosterone treatment increases the risk of de novo prostate cancer, clinical trials have not had enough participants or long enough follow-up to be conclusive. Testosterone stimulates metastatic and high-grade prostate cancer, but it is not known whether testosterone treatment stimulates the growth of pre-existing subclinical lower grade prostate cancer. Testosterone treatment has not been shown to have a significant effect on lower urinary tract symptoms (74); however, clinical trials have generally excluded men with severe benign prostatic hypertrophy. Testosterone should generally not be prescribed for men with prostate cancer (with possible exceptions in carefully selected men such as those with a history of treated low-risk, Gleason <3 + 4 prostate cancer, undetectable PSA and no clinical evidence of disease after treatment) or to men at higher risk of prostate cancer (unevaluated prostate nodule, PSA >4 ng/mL), and follow-up of prostate health during testosterone treatment should be individualized (14). Individualized prostate cancer screening should be offered with a nuanced discussion of the potential benefits (possible early detection of prostate cancer that might improve mortality and decrease the risk of metastatic disease) and the potential harm of increased prostate biopsies and prostate cancer therapies that may cause erectile dysfunction and incontinence and might not always improve long-term, health-related quality of life.

## Back to the Case

The patient has continued to have symptoms suggestive of androgen deficiency and reproducibly low serum testosterone concentrations. He enquired about testosterone therapy to restore his serum testosterone concentration into the normal range. Before testosterone therapy was considered and the potential benefits and risks were discussed, prostate cancer screening was discussed; untreated prostate cancer is a contraindication to testosterone therapy (Table 4). After discussion of the benefits and risks of prostate cancer screening, he elected to undergo prostate cancer screening; digital rectal exam revealed no prostate abnormality and PSA was 1.5 ng/dL. The potential benefits and risks of testosterone therapy were discussed because he had persistent symptoms of hypogonadism and reproducibly, unequivocally low serum testosterone concentrations after a year of addressing reversible causes of a suppressed HPT axis (including lifestyle changes to lose weight and optimization of treatment of sleep apnea).

**Table 4.** Contraindications to testosterone therapy in men

Absolute contraindications	Untreated high-grade prostate cancer	
	Untreated breast cancer	
	Severe lower urinary tract symptoms	
	Unevaluated prostate nodule	
	Idiopathic venous thromboembolism in the past 3 months	
Relative contraindications	Treated Gleason stage 3+4 or 3+3 prostate cancer confined to prostate and in remission	Requires informed consent of patient. Consider consultation with urologist or oncologist.
	Treated breast cancer in remission	
	Recurrent venous thromboembolism or thrombophilia	Hematological assessment and anticoagulation required.
	Erythrocytosis (before initiation of testosterone therapy)	Investigate etiology and hematological assessment as required.
	Major adverse cardiovascular event in the past 6 months	Requires documentation of benefit-risk analysis; Documentation of informed consent recommended.

He was counseled about the potential benefits and risks of testosterone therapy (including the lack of definitive data about the effects on cardiovascular outcomes and prostate cancer) and the absence of high-quality, long-term studies. The patient's main concerns were identified as low libido, reduced energy, and low mood. He was advised that, based on the T-Trials (16), effects of testosterone treatment on these symptoms should be evident within 3 to 6 months while other benefits (eg, muscle mass and function, bone health) typically take longer. Testosterone formulations (including topical and intramuscular treatment) were discussed. He preferred topical testosterone for a therapeutic trial of 6 months to determine if testosterone therapy would meaningfully improve his symptoms. He agreed that testosterone treatment would be stopped if there was no significant benefit despite normal serum testosterone concentrations during therapy. The discussion about the benefits and risks of testosterone, the goals of therapeutic trial and the plan to discontinue testosterone therapy if these goals were not met were documented in the medical record. Treatment was initiated with a standardized monitoring plan (14) that included follow-up clinic visits to assess for effectiveness and safety at 3 and 6 months with reassessment of the dosage of testosterone based on symptoms and measurement of serum testosterone concentrations and assessment of hematocrit 3 and 6 months after initiation (and increases of dosage) of testosterone.

He began transdermal testosterone therapy at a typical replacement dosage, and the dosage was adjusted to achieve a serum total testosterone in the normal range. At 3- and 6-month follow-up evaluations, he reported improved sense of well-being and sexual function. His serum hemoglobin and hematocrit had increased 5% above baseline, but both remained in the normal range. He had lost another 4.3 kg (and now weighs 93.0 kg) with diet and exercise. He had lost a total of 6.3 kg (∼6% of body weight since his initial evaluation). At 12 months of follow-up, he reported that the “testosterone therapy does not seem to be working as well for sexual function.” His bone density (2 years after the last bone densitometry) was not significantly changed.

After discussion, he agreed to discontinue testosterone therapy and reassess serum testosterone. Two months after discontinuation, his serum total testosterone was 260 ng/dL (9.02 nmol/L; reference range, 264-916 ng/dL or 9.16-31.8 nmol/L) in a validated, harmonized assay, and his serum free T was 75 pg/mL (259 pmol/L; reference range, 66-272.3 pg/mL or 229-710 pmol/L). Repeat testing 3 months after discontinuation demonstrated a serum total testosterone of 266 ng/dL (9.2 nmol/L), and a serum calculated free T of 77 pg/mL (267 pmol/L). He noticed no change in symptoms after discontinuation of testosterone therapy. He enquired about a higher dosage of testosterone.

This patient might have had a placebo effect from testosterone therapy that typically abates after ∼ 6 months. Sense of well-being and sexual function are complex, and both depend upon multiple factors beyond serum sex steroid hormone concentrations and the HPT axis. About 60% to 70% of men discontinue testosterone therapy within 12 months of the initial prescription (77, 78); this high discontinuation rate is consistent with the conclusion that most eugonadal men fail to have a clinically significant and durable benefit from testosterone therapy at conventional dosages used for hypogonadal men (90). The high discontinuation rate is also reassuring because most men who meet the criteria for the diagnosis of hypogonadism after attempts to treat reversible causes are likely to stop unbeneficial testosterone therapy, especially if the duration of testosterone treatment is 12 months or less (45, 76). An alternative explanation is that this patient had a benefit in sense of well-being and sexual function that has now been sustained by restoration of normal HPT axis function by weight loss. With either explanation, there is no rationale for continuing testosterone therapy for this man. There is also no evidence of benefit for an increased dosage of testosterone in this clinical setting. Extensive anecdotal clinical experience suggests that most men fail to benefit from an increase in dosage when their current regimen is sufficient to keep their serum testosterone concentration in the normal range.

## Final Comments About the Initiation of Testosterone Therapy in Men ≥50

The decision to treat medical conditions always involves an analysis of the benefits and risks; that analysis is particularly important in the decision to initiate testosterone therapy in men ≥50 years. Although testosterone replacement for men with organic hypogonadism is uncontroversial irrespective of age, men ≥50 years old with nonspecific symptoms, reproducibly low serum testosterone concentrations, normal serum gonadotropins, and no evidence of hypothalamic-pituitary disease often have improved health and well-being and normalization of serum testosterone concentrations with optimization of weight and comorbidities and stopping or reducing the dosage of medications that affect the HPT axis. In addition, therapies other than testosterone should be considered for symptoms and signs that might be attributed to hypogonadism: PDE5-inhibitors (for erectile dysfunction); treatment of sleep apnea, depression, obesity, and diabetes mellitus with proven effective therapies; and medications that reduce fracture (for osteoporosis). It is reasonable to add testosterone treatment to men ≥50 years with persistent symptoms of hypogonadism and reproducibly low serum testosterone (measured fasted in the morning when the man is at baseline health) and normal serum gonadotropin concentrations despite lifestyle measures for 6-12 months to lose weight (if BMI > 25 kg/m^2^) and optimization of management of comorbidities. This decision must be individualized and is not algorithmic, and there should be no contraindications to testosterone therapy. Men ≥50 years with symptoms of hypogonadism and primary hypogonadism (low serum testosterone and elevated serum gonadotropin concentrations) should generally be offered testosterone replacement therapy. If testosterone therapy is being considered, we highlight the importance of discussing the unknown effects of testosterone therapy on the risk of cardiovascular disease and prostate cancer with the patient and documenting that discussion. It is also vital to determine the patient's expectations and goals and to provide information to the patient about the most likely benefits of testosterone therapy and the typical course for these benefits. The clinician and patient should identify patient-specific treatment goals with a timeline for assessment of effectiveness and consideration of whether to continue therapy. The therapeutic plan should include monitoring to ensure serum testosterone concentrations are above the lower limit of normal and to minimize and manage adverse effects including erythrocytosis. If a trial of testosterone therapy of sufficient duration does not meet patient-specific treatment goals in men with low serum testosterone and without identifiable pathology of the HPT axis, testosterone treatment should be stopped.

## Disclosure

M.G. has received research funding from Bayer and Otsuka and speaker's honoraria from Besins Health Care and Novartis. M.G. is funded by an NHMRC project grant (#1099173). C.N.J. is funded by an NIHR Post-Doctoral Fellowship and NIHR Imperial BRC. C.N.J. holds an investigator-led grant from Logixx Pharma Ltd. B.A. is funded as a co-investigator by NIH-NICHD (HHSN275000251) and as the site PI on NIH-RO1HL1343653 (Kanias, PI). B.A. is also a consultant for the United States Anti-Doping Agency.

## Data Availability

Data sharing is not applicable to this article as no datasets were generated or analyzed during the current study.

## Abbreviations

BMD: bone mineral density
BMI: body mass index
CPAP: continuous positive airway pressure
CT: computed tomography
FSH: follicle-stimulating hormone
GLP-1: glucagon-like peptide 1
HPT: hypothalamic-pituitary-testicular (gonadal)
LH: luteinizing hormone
PDE5: phosphodiesterase type 5-inhibitor
RCT: randomized controlled trial
SGLT2: sodium-glucose cotransporter 2
SHBG: sex hormone binding globulin
T2D: type 2 diabetes mellitus

## References

[1] Grossmann M , Matsumoto AM. A perspective on middle-aged and older men with functional hypogonadism: focus on holistic management. J Clin Endocrinol Metab. 2017;102(3):1067‐1075. 28359097 10.1210/jc.2016-3580 PMC5477803

[2] Handelsman DJ . Androgen misuse and abuse. Endocr Rev. 2021;42(4):457‐501. 33484556 10.1210/endrev/bnab001

[3] Thirumalai A , Anawalt BD. Epidemiology of male hypogonadism. Endocrinol Metab Clin North Am. 2022;51(1):1‐27. 35216709 10.1016/j.ecl.2021.11.016 PMC9136962

[4] Anawalt BD , Matsumoto AM. Aging and androgens: physiology and clinical implications. Rev Endocr Metab Disord. 2022;23(6):1123‐1137. 36459352 10.1007/s11154-022-09765-2 PMC10370404

[5] Wu FC , Tajar A, Beynon JM, et al Identification of late-onset hypogonadism in middle-aged and elderly men. N Engl J Med. 2010;363(2):123‐135. 20554979 10.1056/NEJMoa0911101

[6] Camacho EM , Huhtaniemi IT, O’Neill TW, et al Age-associated changes in hypothalamic-pituitary-testicular function in middle-aged and older men are modified by weight change and lifestyle factors: longitudinal results from the European male ageing study. Eur J Endocrinol. 2013;168(3):445‐455. 23425925 10.1530/EJE-12-0890

[7] Yeap BB , Grossmann M, McLachlan RI, et al Endocrine Society of Australia position statement on male hypogonadism (part 1): assessment and indications for testosterone therapy. Med J Aust. 2016;205(4):173‐178. 27510348 10.5694/mja16.00393

[8] Hsu B , Cumming RG, Blyth FM, et al The longitudinal relationship of sexual function and androgen status in older men: the Concord Health and Ageing in Men project. J Clin Endocrinol Metab. 2015;100(4):1350‐1358. 25629357 10.1210/jc.2014-4104

[9] Matsumoto AM . Diagnosis and evaluation of hypogonadism. Endocrinol Metab Clin North Am. 2022;51(1):47‐62. 35216720 10.1016/j.ecl.2021.11.001

[10] Fink HA , Ewing SK, Ensrud KE, et al Association of testosterone and estradiol deficiency with osteoporosis and rapid bone loss in older men. J Clin Endocrinol Metab. 2006;91(10):3908‐3915. 16849417 10.1210/jc.2006-0173

[11] Uddin SMI , Mirbolouk M, Dardari Z, et al Erectile dysfunction as an independent predictor of future cardiovascular events: the Multi-Ethnic Study of Atherosclerosis. Circulation. 2018;138(5):540‐542. 29891569 10.1161/CIRCULATIONAHA.118.033990 PMC6289871

[12] O’Byrne NA , Yuen F, Niaz W, Liu PY. Sleep and the testis. Curr Opin Endocr Metab Res. 2021;18:83‐93. 33937581 10.1016/j.coemr.2021.03.002 PMC8087280

[13] Hoffstein V , Herridge M, Mateika S, Redline S, Strohl KP. Hematocrit levels in sleep apnea. Chest. 1994;106(3):787‐791. 8082360 10.1378/chest.106.3.787

[14] Bhasin S , Brito JP, Cunningham GR, et al Testosterone therapy in men with hypogonadism: an endocrine society clinical practice guideline. J Clin Endocrinol Metab. 2018;103(5):1715‐1744. 29562364 10.1210/jc.2018-00229

[15] Bhasin S , Pencina M, Jasuja GK, et al Reference ranges for testosterone in men generated using liquid chromatography tandem mass spectrometry in a community-based sample of healthy nonobese young men in the Framingham Heart Study and applied to three geographically distinct cohorts. J Clin Endocrinol Metab. 2011;96(8):2430‐2439. 21697255 10.1210/jc.2010-3012 PMC3146796

[16] Snyder PJ , Bhasin S, Cunningham GR, et al Effects of testosterone treatment in older men. N Engl J Med. 2016;374(7):611‐624. 26886521 10.1056/NEJMoa1506119 PMC5209754

[17] Bhasin S , Lincoff AM, Basaria S, et al Effects of long-term testosterone treatment on cardiovascular outcomes in men with hypogonadism: rationale and design of the TRAVERSE study. Am Heart J. 2022;245:41‐50. 34871580 10.1016/j.ahj.2021.11.016

[18] Brambilla DJ , O'Donnell AB, Matsumoto AM, McKinlay JB. Intraindividual variation in levels of serum testosterone and other reproductive and adrenal hormones in men. Clin Endocrinol (Oxf). 2007;67(6):853‐862. 18052942 10.1111/j.1365-2265.2007.02976.x

[19] Sartorius G , Spasevska S, Idan A, et al Serum testosterone, dihydrotestosterone and estradiol concentrations in older men self-reporting very good health: the Healthy Man Study. Clin Endocrinol (Oxf). 2012;77(5):755‐763. 22563890 10.1111/j.1365-2265.2012.04432.x

[20] Snyder PJ , Peachey H, Hannoush P, et al Effect of testosterone treatment on bone mineral density in men over 65 years of age. J Clin Endocrinol Metab. 1999;84(6):1966‐1972. 10372695 10.1210/jcem.84.6.5741

[21] Bremner WJ , Vitiello MV, Prinz PN. Loss of circadian rhythmicity in blood testosterone levels with aging in normal men. J Clin Endocrinol Metab. 1983;56(6):1278‐1281. 6841562 10.1210/jcem-56-6-1278

[22] Anawalt BD , Hotaling JM, Walsh TJ, Matsumoto AM. Performance of total testosterone measurement to predict free testosterone for the biochemical evaluation of male hypogonadism. J Urol. 2012;187(4):1369‐1373. 22341266 10.1016/j.juro.2011.11.095 PMC10368284

[23] Cooper LA , Page ST, Amory JK, Anawalt BD, Matsumoto AM. The association of obesity with sex hormone-binding globulin is stronger than the association with ageing-implications for the interpretation of total testosterone measurements. Clin Endocrinol (Oxf). 2015;83(6):828‐833. 25777143 10.1111/cen.12768 PMC4782930

[24] Goldman AL , Bhasin S, Wu FCW, Krishna M, Matsumoto AM, Jasuja R. A reappraisal of testosterone’s binding in circulation: physiological and clinical implications. Endocr Rev. 2017;38(4):302‐324. 28673039 10.1210/er.2017-00025 PMC6287254

[25] Jasuja R , Pencina KM, Spencer DJ, et al Reference intervals for free testosterone in adult men measured using a standardized equilibrium dialysis procedure. Andrology. 2023;11(1):125‐133. 36251328 10.1111/andr.13310

[26] Hsu B , Cumming RG, Blyth FM, et al Evaluating calculated free testosterone as a predictor of morbidity and mortality independent of testosterone for cross-sectional and 5 year longitudinal health outcomes in older men: the Concord Health and Ageing in Men project. J Gerontol A Biol Sci Med Sci. 2018 May 9;73(6):729‐736. 28958048 10.1093/gerona/glx170

[27] Rastrelli G , O’Neill TW, Ahern T, et al Symptomatic androgen deficiency develops only when both total and free testosterone decline in obese men who may have incident biochemical secondary hypogonadism: prospective results from the EMAS. Clin Endocrinol (Oxf). 2018;89(4):459‐469. 29855071 10.1111/cen.13756

[28] Handelsman DJ . Free testosterone: pumping up the tires or ending the free ride? Endocr Rev. 2017;38(4):297‐301. 28898980 10.1210/er.2017-00171

[29] Anawalt BD . The model T. J Clin Endocrinol Metab. 2016;101(7):2640‐2642. 27381959 10.1210/jc.2016-1820

[30] Iturriaga H , Valladares L, Hirsch S, et al Effects of abstinence on sex hormone profile in alcoholic patients without liver failure. J Endocrinol Invest. 1995;18(8):638‐644. 8655923 10.1007/BF03349782

[31] Iturriaga H , Lioi X, Valladares L. Sex hormone-binding globulin in non-cirrhotic alcoholic patients during early withdrawal and after longer abstinence. Alcohol Alcohol. 1999;34(6):903‐909. 10659727 10.1093/alcalc/34.6.903

[32] Vermeulen A , Verdonck L, Kaufman JM. A critical evaluation of simple methods for the estimation of free testosterone in serum. J Clin Endocrinol Metab. 1999;84(10):3666‐3672. 10523012 10.1210/jcem.84.10.6079

[33] Tajar A , Forti G, O’Neill TW, et al Characteristics of secondary, primary, and compensated hypogonadism in aging men: evidence from the European Male Ageing Study. J Clin Endocrinol Metab. 2010;95(4):1810‐1818. 20173018 10.1210/jc.2009-1796

[34] Yeap BB , Manning L, Chubb SAP, et al Progressive impairment of testicular endocrine function in ageing men: testosterone and dihydrotestosterone decrease, and luteinizing hormone increases, in men transitioning from the 8th to 9th decades of life. Clin Endocrinol (Oxf). 2018;88(1):88‐95. 28945276 10.1111/cen.13484

[35] Grossmann M . Hypogonadism and male obesity: focus on unresolved questions. Clin Endocrinol (Oxf). 2018;89(1):11‐21. 29683196 10.1111/cen.13723

[36] Hirsch D , Benbassat C, Toledano Y, et al Pituitary imaging findings in male patients with hypogonadotrophic hypogonadism. Pituitary. 2015;18(4):494‐499. 25246077 10.1007/s11102-014-0601-x

[37] Chanson P , Raverot G, Castinetti F, et al Management of clinically non-functioning pituitary adenoma. Ann Endocrinol (Paris). 2015;76(3):239‐247. 26072284 10.1016/j.ando.2015.04.002

[38] Molitch ME . Diagnosis and treatment of pituitary adenomas: a review. JAMA. 2017;317(5):516‐524. 28170483 10.1001/jama.2016.19699

[39] Rastrelli G , Carter EL, Ahern T, et al Development of and recovery from secondary hypogonadism in aging men: prospective results from the EMAS. J Clin Endocrinol Metab. 2015;100(8):3172‐3182. 26000545 10.1210/jc.2015-1571

[40] Villareal DT , Chode S, Parimi N, et al Weight loss, exercise, or both and physical function in obese older adults. N Engl J Med. 2011;364(13):1218‐1229. 21449785 10.1056/NEJMoa1008234 PMC3114602

[41] Pattyn N , Cornelissen VA, Eshghi SR, Vanhees L. The effect of exercise on the cardiovascular risk factors constituting the metabolic syndrome: a meta-analysis of controlled trials. Sports Med. 2013;43(2):121‐133. 23329606 10.1007/s40279-012-0003-z PMC3693431

[42] Maio G , Saraeb S, Marchiori A. Physical activity and PDE5 inhibitors in the treatment of erectile dysfunction: results of a randomized controlled study. J Sex Med. 2010;7(6):2201‐2208. 20367777 10.1111/j.1743-6109.2010.01783.x

[43] Corona G , Rastrelli G, Monami M, et al Body weight loss reverts obesity-associated hypogonadotropic hypogonadism: a systematic review and meta-analysis. Eur J Endocrinol. 2013;168(6):829‐843. 23482592 10.1530/EJE-12-0955

[44] Ng Tang Fui M , Prendergast LA, Dupuis P, et al Effects of testosterone treatment on body fat and lean mass in obese men on a hypocaloric diet: a randomised controlled trial. BMC Med. 2016;14(1):153. 27716209 10.1186/s12916-016-0700-9 PMC5054608

[45] Ng Tang Fui M , Hoermann R, Zajac JD, Grossmann M. The effects of testosterone on body composition in obese men are not sustained after cessation of testosterone treatment. Clin Endocrinol (Oxf). 2017;87(4):336‐343. 28561278 10.1111/cen.13385

[46] Armamento-Villareal R , Aguirre LE, Qualls C, Villareal DT. Effect of lifestyle intervention on the hormonal profile of frail, obese older men. J Nutr Health Aging. 2016;20(3):334‐340. 26892583 10.1007/s12603-016-0698-x PMC4811358

[47] Mora M , Aranda GB, de Hollanda A, Flores L, Puig-Domingo M, Vidal J. Weight loss is a major contributor to improved sexual function after bariatric surgery. Surg Endosc. 2013;27(9):3197‐3204. 23612762 10.1007/s00464-013-2890-y

[48] Barnouin Y , Armamento-Villareal R, Celli A, et al Testosterone replacement therapy added to intensive lifestyle intervention in older men with obesity and hypogonadism. J Clin Endocrinol Metab. 2021;106(3):e1096‐e1110. 33351921 10.1210/clinem/dgaa917

[49] Clarke BM , Vincent AD, Martin S, et al Obstructive sleep apnea is not an independent determinant of testosterone in men. Eur J Endocrinol. 2020;183(1):31‐39. 32348955 10.1530/EJE-19-0978

[50] Grunstein RR , Handelsman DJ, Lawrence SJ, Blackwell C, Caterson ID, Sullivan CE. Neuroendocrine dysfunction in sleep apnea: reversal by continuous positive airways pressure therapy. J Clin Endocrinol Metab. 1989;68(2):352‐358. 2493027 10.1210/jcem-68-2-352

[51] Liu PY . A clinical perspective of sleep and andrological health: assessment, treatment considerations, and future research. J Clin Endocrinol Metab. 2019;104(10):4398‐4417. 31042277 10.1210/jc.2019-00683 PMC6735730

[52] Cignarelli A , Castellana M, Castellana G, et al Effects of CPAP on testosterone levels in patients with obstructive sleep apnea: a meta-analysis study. Front Endocrinol (Lausanne). 2019;10:551. 31496991 10.3389/fendo.2019.00551 PMC6712440

[53] Schulz R , Bischof F, Galetke W, et al CPAP therapy improves erectile function in patients with severe obstructive sleep apnea. Sleep Med. 2019;53:189‐194. 29773460 10.1016/j.sleep.2018.03.018

[54] Dhindsa S , Prabhakar S, Sethi M, Bandyopadhyay A, Chaudhuri A, Dandona P. Frequent occurrence of hypogonadotropic hypogonadism in type 2 diabetes. J Clin Endocrinol Metab. 2004;89(11):5462‐5468. 15531498 10.1210/jc.2004-0804

[55] Grossmann M , Thomas MC, Panagiotopoulos S, et al Low testosterone levels are common and associated with insulin resistance in men with diabetes. J Clin Endocrinol Metab. 2008;93(5):1834‐1840. 18319314 10.1210/jc.2007-2177

[56] Jensterle M , Podbregar A, Goricar K, Gregoric N, Janez A. Effects of liraglutide on obesity-associated functional hypogonadism in men. Endocr Connect. 2019;8(3):195‐202. 30707677 10.1530/EC-18-0514 PMC6391904

[57] Bajaj HS , Gerstein HC, Rao-Melacini P, et al Erectile function in men with type 2 diabetes treated with dulaglutide: an exploratory analysis of the REWIND placebo-controlled randomised trial. Lancet Diabetes Endocrinol. 2021;9(8):484‐490. 34153269 10.1016/S2213-8587(21)00115-7

[58] Graybill S , Hatfield J, Kravchenko M, et al Neutral effect of exenatide on serum testosterone in men with type 2 diabetes mellitus: a prospective cohort. Andrology. 2021;9(3):792‐800. 33400403 10.1111/andr.12966

[59] Motta G , Zavattaro M, Romeo F, Lanfranco F, Broglio F. Risk of erythrocytosis during concomitant testosterone and SGLT2-inhibitor treatment: a warning from two clinical cases. J Clin Endocrinol Metab. 2019;104(3):819‐822. 30395251 10.1210/jc.2018-01702

[60] Snyder PJ , Peachey H, Berlin JA, et al Effects of testosterone replacement in hypogonadal men. J Clin Endocrinol Metab. 2000;85(8):2670‐2677. 10946864 10.1210/jcem.85.8.6731

[61] Cunningham GR , Stephens-Shields AJ, Rosen RC, et al Testosterone treatment and sexual function in older men with low testosterone levels. J Clin Endocrinol Metab. 2016;101(8):3096‐3104. 27355400 10.1210/jc.2016-1645 PMC4971331

[62] Corona G , Rastrelli G, Morgentaler A, Sforza A, Mannucci E, Maggi M. Meta-analysis of results of testosterone therapy on sexual function based on international index of erectile function scores. Eur Urol. 2017;72(6):1000‐1011. 28434676 10.1016/j.eururo.2017.03.032

[63] Spitzer M , Basaria S, Travison TG, et al Effect of testosterone replacement on response to sildenafil citrate in men with erectile dysfunction: a parallel, randomized trial. Ann Intern Med. 2012;157(10):681‐691. 23165659 10.7326/0003-4819-157-10-201211200-00004

[64] Diem SJ , Greer NL, MacDonald R, et al Efficacy and safety of testosterone treatment in men: an evidence report for a clinical practice guideline by the American College of Physicians. Ann Intern Med. 2020;172(2):105‐118. 31905375 10.7326/M19-0830

[65] Isidori AM , Giannetta E, Greco EA, et al Effects of testosterone on body composition, bone metabolism and serum lipid profile in middle-aged men: a meta-analysis. Clin Endocrinol (Oxf). 2005;63(3):280‐293. 16117815 10.1111/j.1365-2265.2005.02339.x

[66] Sinclair M , Grossmann M, Hoermann R, Angus PW, Gow PJ. Testosterone therapy increases muscle mass in men with cirrhosis and low testosterone: A randomised controlled trial. J Hepatol. 2016;65(5):906‐913. 27312945 10.1016/j.jhep.2016.06.007

[67] Travison TG , Basaria S, Storer TW, et al Clinical meaningfulness of the changes in muscle performance and physical function associated with testosterone administration in older men with mobility limitation. J Gerontol A Biol Sci Med Sci. 2011;66(10):1090‐1099. 21697501 10.1093/gerona/glr100 PMC3202898

[68] Bhasin S , Ellenberg SS, Storer TW, et al Effect of testosterone replacement on measures of mobility in older men with mobility limitation and low testosterone concentrations: secondary analyses of the testosterone trials. Lancet Diabetes Endocrinol. 2018;6(11):879‐890. 30366567 10.1016/S2213-8587(18)30171-2 PMC6816466

[69] Grossmann M , Hoermann R, Wittert G, Yeap BB. Effects of testosterone treatment on glucose metabolism and symptoms in men with type 2 diabetes and the metabolic syndrome: a systematic review and meta-analysis of randomized controlled clinical trials. Clin Endocrinol (Oxf). 2015;83(3):344‐351. 25557752 10.1111/cen.12664

[70] Wittert G , Bracken K, Robledo KP, et al Testosterone treatment to prevent or revert type 2 diabetes in men enrolled in a lifestyle programme (T4DM): a randomised, double-blind, placebo-controlled, 2-year, phase 3b trial. Lancet Diabetes Endocrinol. 2021;9(1):32‐45. 33338415 10.1016/S2213-8587(20)30367-3

[71] Ng Tang Fui M , Hoermann R, Bracken K, et al Effect of testosterone treatment on bone microarchitecture and bone mineral density in men: a 2-year RCT. J Clin Endocrinol Metab. 2021;106(8):e3143‐e3e58. 33693907 10.1210/clinem/dgab149

[72] Snyder PJ , Kopperdahl DL, Stephens-Shields AJ, et al Effect of testosterone treatment on volumetric bone density and strength in older men with low testosterone: a controlled clinical trial. JAMA Intern Med. 2017;177(4):471‐479. 28241231 10.1001/jamainternmed.2016.9539 PMC5433755

[73] Roy CN , Snyder PJ, Stephens-Shields AJ, et al Association of testosterone levels with anemia in older men: a controlled clinical trial. JAMA Intern Med. 2017;177(4):480‐490. 28241237 10.1001/jamainternmed.2016.9540 PMC5433757

[74] Ponce OJ , Spencer-Bonilla G, Alvarez-Villalobos N, et al The efficacy and adverse events of testosterone replacement therapy in hypogonadal men: a systematic review and meta-analysis of randomized, placebo-controlled trials. J Clin Endocrinol Metab. 2018;103(5):1745‐1754. 10.1210/jc.2018-00404 29562341

[75] Handelsman DJ , Desai R, Conway AJ, et al Recovery of male reproductive endocrine function after ceasing prolonged testosterone undecanoate injections. Eur J Endocrinol. 2022;186(3):307‐318. 35000898 10.1530/EJE-21-0608

[76] Bebb RA , Anawalt BD, Christensen RB, Paulsen CA, Bremner WJ, Matsumoto AM. Combined administration of levonorgestrel and testosterone induces more rapid and effective suppression of spermatogenesis than testosterone alone: a promising male contraceptive approach. J Clin Endocrinol Metab. 1996;81(2):757‐762. 8636300 10.1210/jcem.81.2.8636300

[77] Schoenfeld MJ , Shortridge E, Cui Z, Muram D. Medication adherence and treatment patterns for hypogonadal patients treated with topical testosterone therapy: a retrospective medical claims analysis. J Sex Med. 2013;10(5):1401‐1409. 23464534 10.1111/jsm.12114

[78] Donatucci C , Cui Z, Fang Y, Muram D. Long-term treatment patterns of testosterone replacement medications. J Sex Med. 2014;11(8):2092‐2099. 24909541 10.1111/jsm.12608

[79] Yeap BB , Dwivedi G, Chih HJ, Reid C. Androgens and cardiovascular disease in men. Updated December 14, 2022. In: Feingold KR, Anawalt B, Boyce A, et al, eds. Endotext [Internet]. MDText.com, Inc.; 2000. Accessed January 2023. https://www.ncbi.nlm.nih.gov/sites/books/NBK279151/

[80] Sharma R , Oni OA, Gupta K, et al Normalization of testosterone level is associated with reduced incidence of myocardial infarction and mortality in men. Eur Heart J. 2015;36(40):2706‐2715. 26248567 10.1093/eurheartj/ehv346

[81] Baillargeon J , Urban RJ, Kuo YF, et al Risk of myocardial infarction in older men receiving testosterone therapy. Ann Pharmacother. 2014;48(9):1138‐1144. 24989174 10.1177/1060028014539918 PMC4282628

[82] Vigen R , O'Donnell CI, Baron AE, et al Association of testosterone therapy with mortality, myocardial infarction, and stroke in men with low testosterone levels. JAMA. 2013;310(17):1829‐1836. 24193080 10.1001/jama.2013.280386

[83] Basaria S , Coviello AD, Travison TG, et al Adverse events associated with testosterone administration. N Engl J Med. 2010;363(2):109‐122. 20592293 10.1056/NEJMoa1000485 PMC3440621

[84] Srinivas-Shankar U , Roberts SA, Connolly MJ, et al Effects of testosterone on muscle strength, physical function, body composition, and quality of life in intermediate-frail and frail elderly men: a randomized, double-blind, placebo-controlled study. J Clin Endocrinol Metab. 2010;95(2):639‐650. 20061435 10.1210/jc.2009-1251

[85] Hudson J , Cruickshank M, Quinton R, et al Adverse cardiovascular events and mortality in men during testosterone treatment: an individual patient and aggregate data meta-analysis. Lancet Healthy Longev. 2022;3(6):e381‐ee93. 35711614 10.1016/S2666-7568(22)00096-4 PMC9184259

[86] Budoff MJ , Ellenberg SS, Lewis CE, et al Testosterone treatment and coronary artery plaque volume in older men with low testosterone. JAMA. 2017;317(7):708‐716. 28241355 10.1001/jama.2016.21043 PMC5465430

[87] Basaria S , Harman SM, Travison TG, et al Effects of testosterone administration for 3 years on subclinical atherosclerosis progression in older men with low or low-normal testosterone levels: a randomized clinical trial. JAMA. 2015;314(6):570‐581. 26262795 10.1001/jama.2015.8881

[88] Baillargeon J , Urban RJ, Morgentaler A, et al Risk of venous thromboembolism in men receiving testosterone therapy. Mayo Clin Proc. 2015;90(8):1038‐1045. 26205547 10.1016/j.mayocp.2015.05.012

[89] Martinez C , Suissa S, Rietbrock S, et al Testosterone treatment and risk of venous thromboembolism: population based case-control study. BMJ. 2016;355:i5968. 27903495 10.1136/bmj.i5968 PMC5130924

[90] Bandari J , Ayyash OM, Emery SL, Wessel CB, Davies BJ. Marketing and testosterone treatment in the USA: a systematic review. Eur Urol Focus. 2017;3(4-5):395‐402. 29174614 10.1016/j.euf.2017.10.016

